# Silencing of ETS1 reverses adriamycin resistance in MCF-7/ADR cells via downregulation of MDR1

**DOI:** 10.1186/1475-2867-14-22

**Published:** 2014-03-07

**Authors:** Jinrong Wei, Yong Zhou, Guo-Qin Jiang, Dong Xiao

**Affiliations:** 1 Department of General Surgery, The Second Affiliated of Hospital of Soochow University, Suzhou, Jiangsu 215004, China; 2 Department of General Surgery, Yancheng City No. 1 People’s Hospital, Yancheng, Jiangsu 224005, China; 3 Department Urology, University of Pittsburgh Cancer Institute, University of Pittsburgh Medical College, University of Pittsburgh, Shadyside Medical Center, Suit G37, 5200 Center Avenue, Pittsburgh, PA 15232, USA

**Keywords:** ETS1, Adriamycin, Multidrug resistance, MDR1, Drug efflux, Breast cancer

## Abstract

**Background:**

Clinical resistance to chemotherapeutic agents is one of the major hindrances in the treatment of human cancers. Erythroblastosis virus E26 oncogene homolog 1 (ETS1) is involved in the drug resistance of various cancer cells, and is overexpressed in drug-resistant human breast cancer cell lines. In this study, we investigated the effects of ETS1 on adriamycin resistance in MCF-7/ADR cells.

**Methods:**

siRNAs against ETS1 or negative control siRNAs was transfected to MCF-7/ADR breast cancer cells. Reverse transcription-PCR and Western blotting were used to determine the mRNA and protein expression of ETS1 and MDR1. The cytotoxicity of adriamycin was assessed using the MTT assay. Drug efflux was investigated by flow cytometry using the Rhodamine 123 intracellular accumulation assay.

**Results:**

ETS1 mRNA and protein was significantly overexpressed in MCF-7/ADR cells, compared to MCF-7 cells. ETS1 siRNA successfully silenced ETS1 mRNA and protein expression. Silencing of ETS1 also significantly reduced the mRNA and protein expression levels of MDR1 (multidrug resistance 1; also known as *ABCB1*, P-glycoprotein/P-gp), which is a major ATP-binding cassette (ABC) transporter linked to multi-drug resistance in cancer cells. Silencing of ETS1 significantly increased the sensitivity of MCF-7/ADR cells to adriamycin, compared to cells transfected with negative control siRNA. In addition, intracellular accumulation of Rhodamine 123 significantly increased in MCF-7/ADR cells transfected with ETS1 siRNA, indicating that silencing of ETS1 may reduce drug efflux.

**Conclusions:**

This study demonstrates that drug resistance can be effectively reversed in adriamycin-resistant breast carcinoma cells through delivery of siRNAs targeting ETS1.

## Introduction

Breast cancer is a systemic disease, with the primary tumor representing the localized form of the disease. Therefore, systemic adjuvant therapies play an important role in the treatment of breast cancer, and adjuvant chemotherapy is a central component of systemic adjuvant therapy for breast cancer. However, the resistance of tumor cells to multiple chemotherapeutic agents is a major obstacle to the success of cancer chemotherapy, and has been strongly associated with treatment failure [1]. Multidrug resistance (MDR) refers to the resistance of tumors to not only a single cytotoxic drug, but cross-resistance to a wide range of drugs with unrelated function and structure [2].

The mechanisms of drug resistance are complicated, and include decreased intracellular drug accumulation, which can occur due to increased drug efflux by the ATP-binding cassette (ABC) transporters such as multidrug resistance 1 (MDR1; also known as *ABCB1*, P-glycoprotein/P-gp). A number of important anticancer agents derived from natural products, such as vincaalkaloids, anthracyclines (daunorubicin, adriamycin) and taxanes are substrates of MDR1. Other mechanisms of drug resistance include decreased conversion of a drug to its active form, altered expression of the target enzyme or receptor, decreased affinity of the target enzyme or receptor for the drug, enhanced repair of drug-induced defects, altered expression of apoptosis/survival genes [3].

Reversal of MDR has been the focus of medical studies for many years. Previous research reported that down-regulation of the ABC family members or altering the expression of apoptosis/survival genes can reverse MDR in cancer cells [4,5]. In the past two decades, there has been a worldwide effort to investigate a large number of diverse chemical agents for their ability to overcome MDR. Although these chemical agents are effective in cell culture and animal models, they usually fail in the clinical setting. The reasons for this high rate of failure include the necessity of extremely high inhibitor concentrations which induce unwanted side effects, and unpredictable pharmacokinetic interactions with therapeutic anticancer agents [6].

RNA interference (RNAi) technology provides a novel therapeutic approach for the treatment of drug-resistant tumors. Various RNAi strategies have been applied to reverse MDR in different tumor models *in vitro* and *in vivo* by down-regulating genes associated with MDR, such as multidrug resistance 1(MDR1), multidrug resistance-associated protein(*MRP*) and breast cancer resistance protein (BCRP) [7-9]. Recently, it was reported that Erythroblastosis virus E26 oncogene homolog 1 (ETS1) gene is over-expressed in drug-resistant human breast cancer cell lines [10]. Experimental studies have shown that *ETS1* is involved in the drug resistance of ovarian and pancreatic cancer cells [11,12]. Based on these observations, we hypothesized that down-regulation of ETS1 using siRNAs would result in heightened drug sensitivity and reverse MDR in breast cancer cells. In this study we investigated the effects of ETS1 on adriamycin resistance in MCF-7/ADR cells, which are typical multidrug-resistant human breast cancer cells that were selected by exposure to adriamycin [13].

## Materials and methods

### Cell lines and culture

Human MCF-7 and MCF-7/ADR breast cancer cell lines were obtained from XiangYa Central Experiment Laboratory (Changsha, China), and maintained in RPMI1640 medium (GIBCO, Grand Island, NY, USA) supplemented with 10% fetal bovine serum (FBS), penicillin, and streptomycin at 37°C in 5% CO_2_ as described by us previously [14].

### Synthesis of siRNAs

A double-stranded siRNA oligo nucleotide targeting ETS1 (sense, 5′-ACUUGCUACCAUCCCGUAC-dTT-3′; antisense, 5′-GUACGGGAUGGUAGCAAGU-dTT-3′) was designed based on Ito et al. [15] and synthesized by Shanghai Genepharma Co. Ltd. (China). A pair of negative control siRNAs were also designed by varying the sequence of siRNA-ETS1; the negative control siRNAs were not homologous to any known sequences in GenBank (sense, 5′-UUCUCCGAACGUGUCACGUTT-3′; antisense, 5′-ACGUGACACGUUCGGAGAATT-3′). The siRNAs were dissolved in siRNA dilution buffer (Shanghai Genepharma Co. Ltd. China) to a final concentration of 20 μmol/L.

### RT-PCR analysis

Total cellular RNA was isolated using TRIzol (Invitrogen) according to the manufacturer’s instructions. For reverse transcription (RT)-PCR, 5 μg of total RNA per sample was reverse transcribed using the Reverse Transcription Reaction Kit (Fermentas, St. Leon-Rot, Germany) according to the manufacturer’s instructions. The cDNA (1 μl) was amplified by PCR (pre-denaturation step at 95°C for 5 min; followed by 40 cycles of 95°C for 30 s, 60°C for 30 s, and 72°C for 30 s; then 72°C for 10 min). The primers were as follows: ETS1, 5′-TTCCACCATCGGAGTTCTCAGA-3′ and 5′-GAAGCTGTCATAGGAGGGAACA-3′; MDR1, 5′-TGACTACCAGGCTCGCCAATGAT-3′ and 5′-TGTGCCACCAAGTAGGCTCCAAA-3′; *GAPDH*, 5′-TCCTGTGGCATCCACGAAACT-3′ and 5′-GAAGCATTTGCGGTGGACGAT-3′. The reaction products were visualized by electrophoresis on 1.5% agarose gels using 1xTBE buffer containing 0.5 μg/ml ethidium bromide. The final, normalized results were calculated by dividing the relative transcript levels of the target genes by the relative transcript levels of GAPDH.

### Western blotting

Western blotting was performed as described in our previous study [14]. Briefly, cells were harvested and rinsed with PBS. Cell extracts were prepared using lysis buffer (containing 8 M urea, 10% SDS, 1 M DTT and protease inhibitors) and centrifuged at 12,000 g at 4°C. Total protein concentration was measured using the BCA assay. Cellular extracts containing 30 μg total protein were electrophoresed on 10% SDS-PAGE gels and then transferred onto polyvinylidene difluoride membranes (Invitrogen). The membranes were incubated for 2 h in blocking solution containing 5% non-fat dry milk to inhibit non-specific binding, then incubated with primary anti-ETS-1 (1:2000; Epitomics Inc., Burlingham, CA, USA) and anti-β-actin FLAG (1:5000; Abcam, Cambridge, MA, USA) antibodies for 2 h. After several washes in PBS, the membranes were incubated with HRP-conjugated secondary antibodies (1:3000; Abcam, Cambridge, United Kingdom). The blots were developed using an ECL chemiluminescent kit (Beyotime, Haimen, China), and exposed to X-ray film for 30 s to 2 min.

### Transfection of siRNAs

MCF-7/ADR cells (3 × 10^5^) were plated into the 35 mm wells of 6-well plates and allowed to adhere for 24 h. Prior to transfection, 5 μl of Lipofectamine™ 2000 transfection reagent (Invitrogen, Carlsbad, CA, USA) and 5 μl of siRNA solution was added to Buffer EC-R (Qiagen, Hilden, Germany), to prepare a total volume of 500 μl per well. The complex was gently mixed, incubated at room temperature for 20 min, and added to the wells containing 2 ml DMEM without 10% FBS, after 4-6 h, then the cells were incubated using normal cell culture conditions. Untransfected control and negative control cells (negative control siRNA) were prepared in parallel.

### Cytotoxicity assay

Cell proliferation was determined using the MTT assay. Cells were seeded at 1×10^4^/well in 96-well microtiter plates. After 24 h incubation, the cells were transfected with siRNAs, incubated for 5 h, and then treated with different concentrations of adriamycin (Huasu Pharmaceuticals Inc, Beijing, China) for 48 h. Then, 20 μl of 5 mg/ml MTT solution was added, incubated for 4 h at 37˚C, the medium and MTT solution were discarded, DMSO (150 μl) was added to each well, and the plates were shaken for 30 min. The optical density (OD) values were read using a Synergy HT multi-detection microplate reader (Bio-Tek Instruments, Inc., Winooski, VT, USA) at λ = 570 nm. Relative drug resistance was determined by comparing the IC_50_ values (drug concentration causing a 50% inhibition of cell growth, determined from the growth inhibition curves) for each group.

### Intracellular Rhodamine 123 accumulation assay

The fluorescence intensity of intracellular Rhodamine (Rh123) accumulation was determined by flow cytometry according to standard procedures [3,16]. Cells were plated at 2×10^5^/well in 6-well plates, incubated for 24 h, transiently transfected with siRNAs and then incubated for 48 h. Subsequently, Rh123 was added at a final concentration of 10 μg/ml, the cells were incubated for 1 h, harvested, washed twice with cold PBS, and then placed in iced-water until analysis. After half an hour, the fluorescence intensity of the cells was determined using the FACS Calibur™ flow cytometer (Becton Dickinson, San Jose, CA. USA) at an excitation wavelength of 488 nm and a receiving wavelength of 575 nm.

### Statistical analysis

All RT-PCR and Western blotting data were normalized to *GAPDH* or β-actin, respectively. Statistical analysis was performed using SPSS 13.0 (SPSS, Chicago, IL, USA). All data are presented as the mean ± standard deviation and one-way ANOVA and Dunnett’s T3 post test was used to determine the statistical significance. Differences between groups were analyzed using two-sided t-tests. *P* <0.05 was considered statistically significant.

## Results

### ETS1 is up-regulated in MCF-7/ADR cells compared to MCF-7 cells

**Figure 1:**
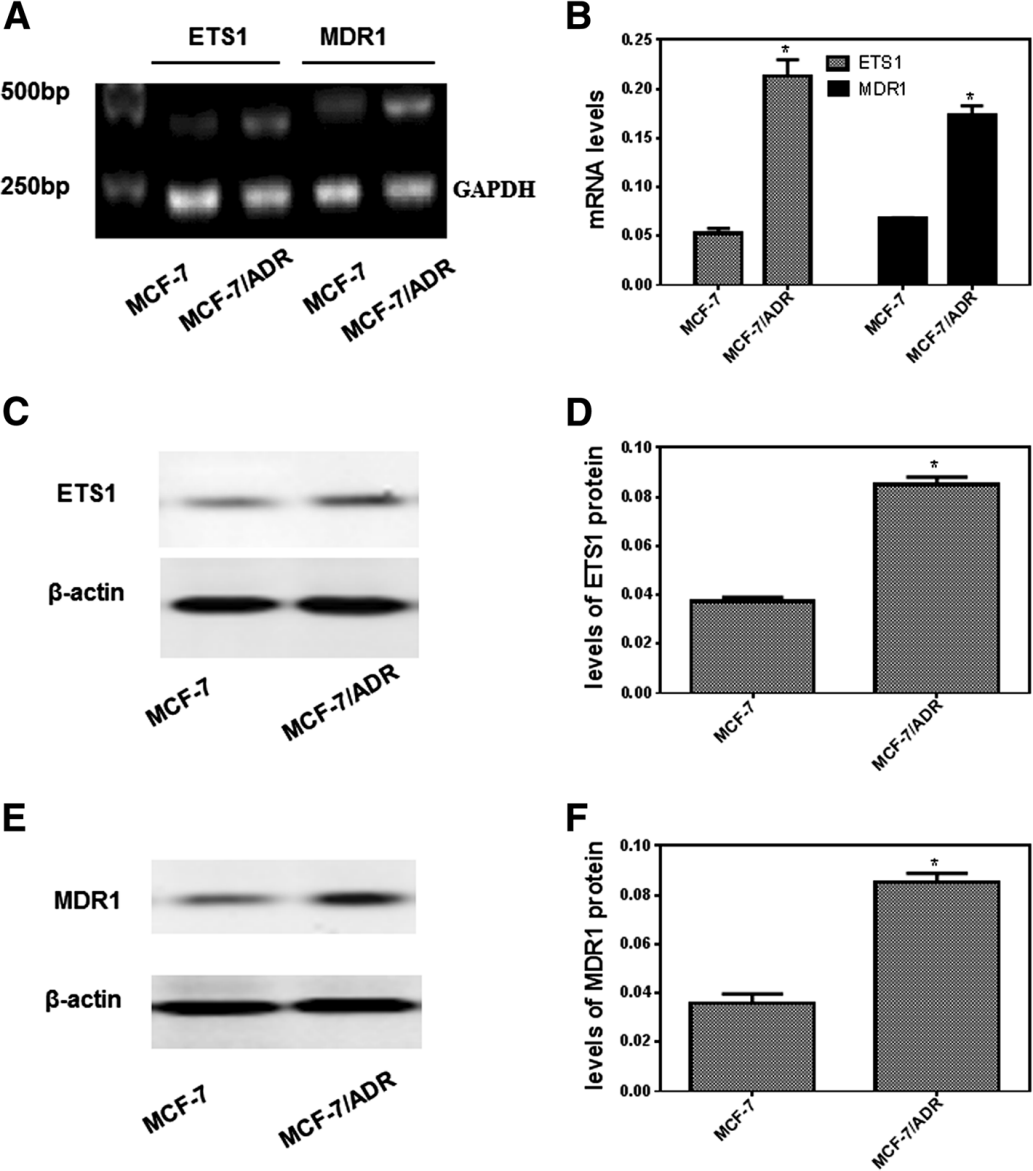
**The levels of mRNA and protein expression of ETS1 and MDR1 in MCF-7 and MCF-7/ADR cells. (A)** RT-PCR analysis and **(B)** quantification of ETS1 and MDR1 mRNA expression in MCF-7 and MCF-7/ADR cells (MCF-7/ADR), the mRNA levels were normalized to the loading control *GAPDH*. The ratio of ETS1 and MDR1 to *GAPDH* mRNA was calculated for each group. Data are mean ± SD of three independent experiments; *P < 0.05 compared with MCF-7 cells. Immunoblotting analysis **(C and E)** and quantification **(D and F)** of ETS1 **(C and D)** and MDR1 **(E and F)** protein expression MCF-7 and MCF-7/ADR cells. The blots were stripped and reprobed with anti-β-actin antibody to ensure equal protein loading. The ratio of ETS1 to β-actin protein was calculated for each group. Values are mean ± SD of three independent experiments; *P < 0.05 compared with MCF-7 cells.

Initially, we determined the mRNA and protein expression of MDR1 in the MCF-7 and MCF-7/ADR cells to confirm the Adriamycin-resistance. The levels of mRNA and protein of MDR1 were highly increased in the MCF-7/ADR cells as compared with the MCF-7 cells (Figure 1A,B,E and F). The expression of ETS1 mRNA in MCF-7 and MCF-7/ADR cells was determined by RT-PCR. The size of the PCR products for ETS1 and *GAPDH* were 345 bp and 225 bp, respectively. As shown in Figure 1, the expression of ETS1 mRNA in MCF-7/ADR cells was 4.1-fold higher than the levels in parental MCF-7 cells (*P* < 0.05). Next, we tested whether the ETS1 protein is up-regulated in MCF-7/ADR cells. As shown in Figure 1C and D, the level of ETS1 in MCF-7/ADR cells was significantly higher than that of MCF-7 cells. These observations clearly indicated that ETS1 is over-expressed in MCF-7/ADR cells (Figure 1).

### Confirmation of ETS1 silencing in ETS1 siRNA-transfected MCF-7/ADR cells

**Figure 2:**
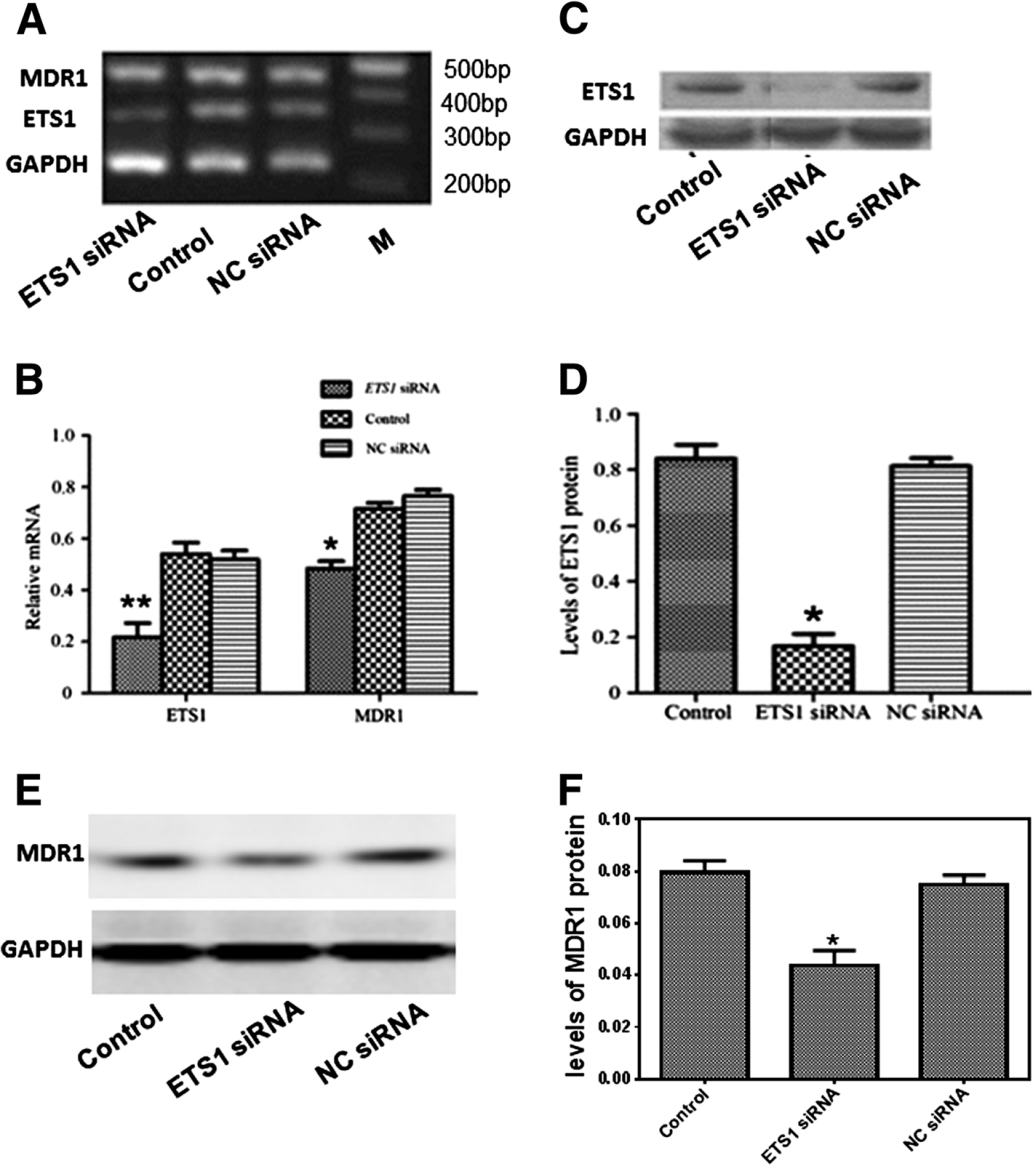
**Silencing of ETS1 reduces the mRNA and protein expression of ETS1 and MDR1 in MCF-7/ADR cells. (A)** RT-PCR analysis and **(B)** quantification of ETS1 and MDR1 mRNA expression in: ETS1 siRNA-transfected cells (ETS1 siRNA); control untransfected cells (Control); and negative control siRNA transfected cells (NC siRNA) at 48 h after transfection. GAPDH was analyzed in parallel as a loading control; the ratio of ETS1 to GAPDH mRNA was calculated for each group. Western blot analysis **(C and E)** and quantification **(D and F)** of ETS1 **(C and D)** and MDR1 **(E and F)** protein expression in: ETS1 siRNA-transfected cells (ETS1 siRNA); control untransfected cells (Control); and negative control siRNA transfected cells (NC siRNA) at 48 h after transfection, β-actin was analyzed in parallel as a loading control; the ratio of ETS1and MDR1 to β-actin protein was calculated for each group. Values are mean ± SD of three independent experiments; *P < 0.05 and **P < 0.01 compared with untransfected control cells.

To confirm the RNAi-mediated silencing of ETS1 in MCF-7/ADR cells, ETS1 mRNA and protein expression were quantified by RT-PCR and Western Blotting respectively, 48 h after transfection of ETS1 siRNA. As shown in Figure 2A, the PCR products for ETS1 and *GAPDH* were 345 bp and 225 bp respectively. As shown in Figure 2B, the expression of ETS1 mRNA declined to 60.1% in the siRNA transfected cells, compared to the negative control cells (*P* < 0.05); there were no significant differences between the untransfected control and negative control transfected cells. Western blot analysis confirmed the results of the RT-PCR analysis (Figure 2C). As shown Figure 2D, ETS1 protein expression was reduced by more than 85% in the siRNA-transfected MCF-7/ADR cells, compared to the negative control transfected cells; there was no significant difference between the untransfected cotrol cells and negative control transfected MCF-7/ADR cells.

### Silencing of ETS1 down-regulated MDR1 mRNA and protein expression in MCF-7/ADR cells

Next, we investigated whether silencing of ETS1 can reduced the expression of MDR1 mRNA and protein in MCF-7/ADR cells. To answer the question, we determined the levels of MDR1 mRNA and protein in the control and transfected MCF-7/ADR cells. The expression of MDR1 mRNA was determined in MCF-7/ADR cells by RT-PCR 48 h after transfection of ETS1 siRNA. The PCR products for MDR1 and *GAPDH* were 457 bp and 225 bp, respectively. As shown Figure 2B, siRNA-mediated silencing of ETS1 reduced the expression of MDR1 mRNA to 67.4% of the levels observed in untransfected control MCF-7/ADR cells (*P* < 0.05); there was no significant difference between the untransfected control cells and negative control transfected MCF-7/ADR cells. More importantly, the ETS1 siRNA-transfected MCF-7/ADR cells resulted in a remarkably decrease of the protein levels of compared to that in control and NC siRNA-transfected MCF-7/ADR cells (Figure 2E and F). For example, the MDR1 level in ETS siRNA-transfected MCF-7/ADR cells was decreased by about 50 %, as compared to the levels in the control group (Figure 2E and F). These observations clearly indicated that silencing of ETS1 resulted in the down-regulation of MDR1 signaling in human breast cancer MCF-7/ADR cells.

### Silencing of ETS1 restored the chemosensitivity of MCF-7/ADR cells

**Figure 3:**
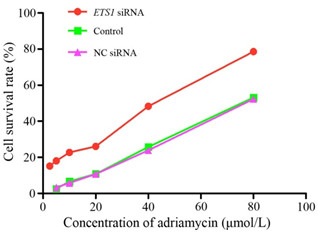
**Silencing of ETS1 reduces the cytotoxicity of adriamycin in MCF-7/ADR cells.** Cells were untransfected (Control), or transfected with EST1 siRNA (ETS1 siRNA) or negative control siRNA (NC siRNA), and then incubated with different concentrations of adriamycin, as indicated for an additional 48 h. Cell survival was determined using the MTT assay. Data are mean ± SD of three independent experiments; *P < 0.05 compared with untransfected control cells.

Then, we determined the effect of silencing of ETS1 on the chemosensitivity of MCF-7/ADR cells by Adriamycin. The IC_50_ for adriamycin in MCF-7/ADR cells was 81.37 ± 6.34 μmol/L at 48 h. As shown Table 1 and Figure 3, transfection of ETS1 siRNA significantly decreased the IC_50_ for adriamycin in MCF-7/ADR cells, compared to untransfected control cells (*P* < 0.05); there was no significant difference between untransfected control cells and negative control transfected MCF-7/ADR cells. These results suggested that MCF-7/ADR cells could be effectively chemosensitized by siRNA-mediated silencing of ETS1.

**Table 1:** **IC**_**50 **_**values for adriamycin in MCF-7/ADR cells**

**Group**	**IC**_**50 **_**(μmol/L)**	**RR**
*EST1* siRNA	34.63 ± 1.29*	2.35
Negative control siRNA	86.18 ± 5.29	0.94
Control untransfected	81.37 ± 6.34	1.00

Cytotoxicity was assayed as described in methods. IC_50_ is the drug concentration (μmol/L) that resulted in a 50% inhibition of cell growth. The RR (relative drug resistance) of the control cells was set as 1. Results are mean ± SD of triplicate determinations; *P < 0.05 compared with the controls.

### Drug efflux is reduced in ETS1 siRNA -transfected MCF-7/ADR cells

**Figure 4:**
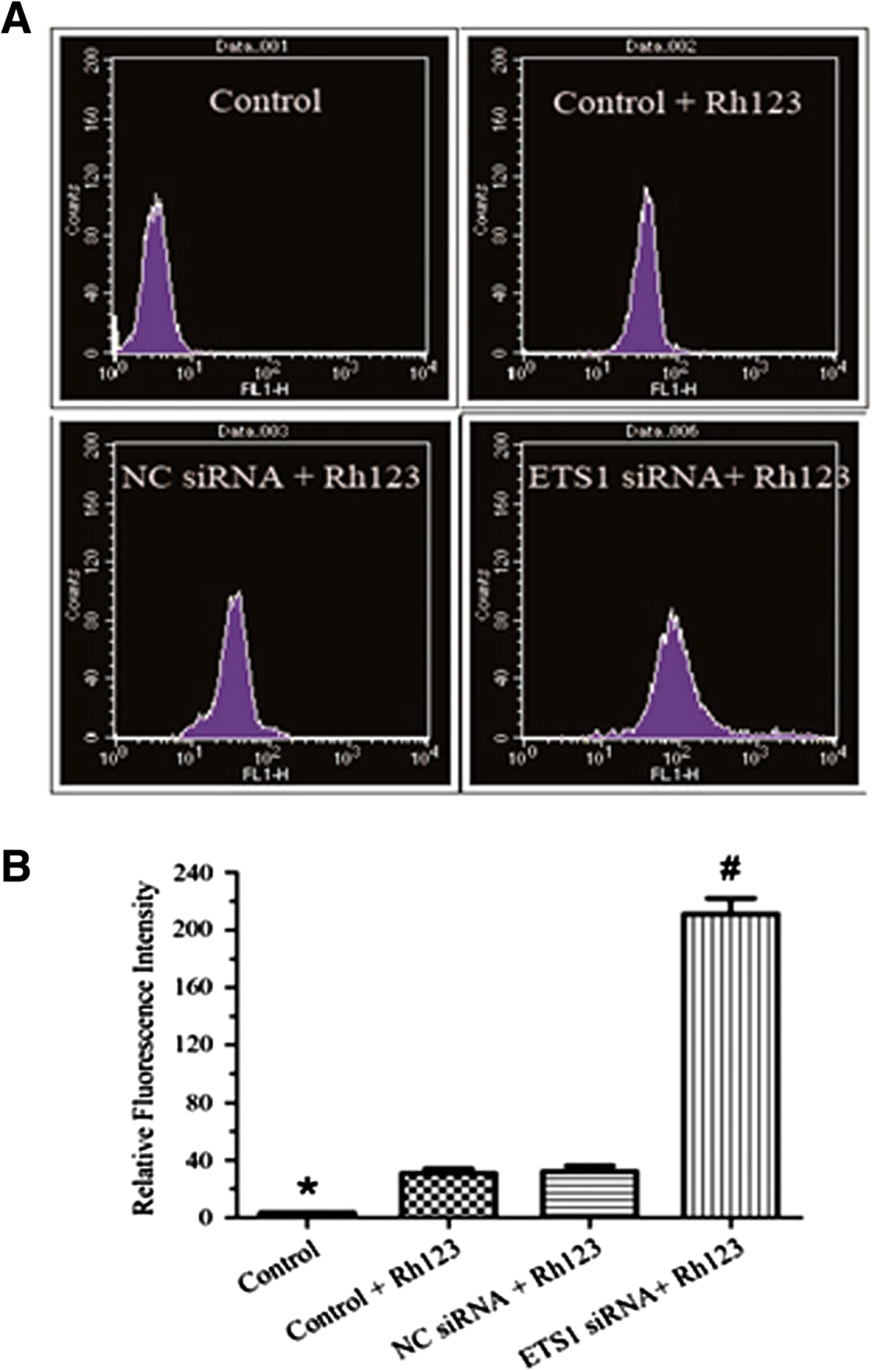
**Rhodamine 123 (Rh123) efflux assay. (A)** MDR1-mediated rhodamine efflux was measured by flow cytometric analysis of Rh123 intracellular accumulation. Flow cytometry analysis and **(B)** mean fluorescence intensity of control untransfected MCF-7/ADR cells (Control); control untransfected MCF-7/ADR cells treated with Rh123(control +Rh123); negative control siRNA transfected MCF-7/ADR cells treated with Rh123(NC siRNA + Rh123); and *ETS1* siRNA-transfected MCF-7/ADR cells treated with Rh123(ETS1 siRNA+ Rh123). Results are mean ± SD of three independent experiments. *P < 0.05 compared with MCF-7/ADR cells treated with Rh123; # *P* < 0.01 compared with untransfected MCF-7/ADR cells.

Adriamycin is transported out of the cell by MDR1 (also known as ABCB1 and P-gp). As shown Figure 4A, flow cytometry analysis demonstrated that treatment of MCF-7/ADR cells with Rh123 significantly increased the number of fluorescent cells, compared to control MCF-7/ADR cells. Moreover, the fluorescence intensity of Rh123, a measure of intracellular Rh123 accumulation, was significantly higher in ETS1 siRNA-transfected MCF-7/ADR cells (211.26±19.15) than negative control cells (36.78±3.71; *P* < 0.01; Figure 4B and Table 2). There was no significant difference in intracellular Rh123, accumulation in untransfected control cells and negative control-transfected MCF-7/ADR cells.

**Table 2:** Fluorescence intensity values in the Rh123 intracellular accumulation assay

**Group**	**Fluorescence intensity**
MCF-7/ADR	3.45±0.08*
Control untransfected + Rh123	36.62±3.68
Negative control siRNA + Rh123	36.78±3.71
ETS1 siRNA + Rh123	211.26±19.15^#^

*P < 0.05 compared with the MCF-7/ADM cells treated with Rh123; #*P* < 0.01 compared with the untransfected controls + Rh123.

## Discussion

Chemotherapy is used to treat all stages of breast cancer; however, MDR severely limits the effects of chemotherapy. Recently, it was reported that ETS1 gene is overexpressed in drug-resistant human breast cancer cell lines [10]. MCF-7/ADR cells are typical multidrug-resistant human breast cancer cells, which were selected by exposure to adriamycin [13]. In this study, we confirmed that ETS1 mRNA and protein are overexpressed in adriamycin-resistant human MCF-7/ADR breast cancer cells.

To further investigate the effects of ETS1 on adriamycin resistance in breast cancer, siRNAs targeting ETS1 were transfected into MCF-7/ADR cells. ETS1 siRNAs significantly suppressed the expression of ETS1 in MCF-7/ADR cells, and reduced ETS1 mRNA and protein expression by more than 60% and 85%, respectively, compared to cells transfected with a negative control siRNA. Using siRNAs against ETS1*,* Khanna et al. demonstrated a reversal in gemcitabine chemosensitivity in gemcitabine-resistant cells [12]. We observed a similar reversal in adriamycin chemosensitivity using siRNAs against ETS1 in MCF-7/ADR cells. Silencing of ETS1 significantly decreased the IC_50_ value for adriamycin in MCF-7/ADR cells, indicating that silencing of ETS1 restored the chemosensitivity of MCF-7/ADR cells. MCF-7/ADR cells display an ATP-dependent reduction in the intracellular accumulation of anthracyclines, despite the absence of over-expression of MDR1 (also known as P-glycoprotein/P-gp/ABCB1). A number of notable, anticancer agents derived from natural products, such as vinca alkaloids, anthracyclines (daunorubicin, adriamycin) and taxanes are substrates of MDR1 [6,17,18]. In this study, semi-quantitative RT-PCR analysis indicated that silencing of ETS1 significantly reduced the expression of MDR1 mRNA by more than 35% in MCF-7/ADR cells. The intracellular Rh123 accumulation assay revealed that drug efflux significantly reduced in MCF-7/ADR cells transfected with ETS1 siRNA, demonstrating that silencing of ETS1 downregulated MDR1, which in turn increased accumulation of adriamycin by reducing the ability of MDR1/P-glycoprotein to transport the drug out of the cell. This data is in good agreement with one of the major mechanisms of drug resistance in cancer cells - decreased intracellular drug accumulation, which occurs due to increased drug efflux by the ATP-binding cassette (ABC) transporters such as MDR1/P-glycoprotein [16].

Our findings indicate that ETS1 can regulate the expression of MDR1 and, in turn, the function of MDR1/P-glycoprotein. Wilson et al. previously reported that ETS1 elicited cisplatin resistance via transcriptional activation of genes whose products have well-described functions in reducing the toxicity of cisplatin [11]. Taken together, this evidence indicates that the proto-oncoprotein ETS1 plays an important role in tumor chemoresistance via a number of different mechanisms.

In addition to promoting MDR in cancer cells, ETS1 is also likely to play a role in tumor progression. ETS1 is a member of the ETS family of transcription factors, which share a unique DNA binding domain [19]. ETS1 is expressed by a variety of solid tumors, including epithelial carcinomas, sarcomas and astrocytomas. In some tumor types, expression of ETS1 is either upregulated or observed exclusively in invasive, higher grade tumors. High ETS1 levels correlate with poor prognosis in cancer of the breast, ovary and cervix [20-22]. Previous research reported that cells over-expressing MDR1/P-glycoprotein have increased invasive and metastatic behavior [23,24]. ETS1 regulates a number of genes coding for proteases, such as matrix metalloproteinase-1 (*MMP1*), *MMP3* and *MMP9*, and urokinase type plasminogen activator (*PLAU*). These proteases are known to be involved in extracellular matrix (ECM) degradation, a key event required for the invasion of cancer cells [25,26].

It is notable that ETS1 interacts with mutant P53, but not wild-type P53, which can lead to selective up-regulation of the MDR1 gene *in vitro* and *in vivo*[27]. It is known that some invasive breast cancer cells express mutant P53 [28]; the frequency of P53 mutations in breast cancer is approximately 50% [29]. Our results demonstrate that silencing of ETS1 reduced the expression of MDR1 mRNA less efficiently than the expression of ETS1 mRNA, whereas close to the frequency of P53 mutations. This indicates that ETS1 may not be the only factor which regulates the expression of MDR1. Further research is required to examine the mechanisms regulating MDR1 and its down-stream effectors in ETS1 siRNA transfected cells. This may help to reveal the mechanisms which regulate MDR1 in cells without P53 mutations.

## Abbreviations

EST1: Erythroblastosis virus E26 oncogene homolog 1; siRNAs: small interfering RNA; MDR1: multidrug resistance 1; P-gp: P-glycoprotein; MTT: 3-(4,5-dimethylthiazol-2-yl)-2,5-diphenyltetrazolium bromide; ABC: ATP-binding cassette; RNAi: RNA interference; MDR: Multidrug resistance; MRP: Multidrug resistance-associated protein; BCRP: Breast cancer resistance protein; FBS: Fetal bovine serum; PBS: Phosphate buffered saline; BCA: Bicinchoninic acid; SDS-PAGE: Substrate-sodium dodecyl sulfate-polyacrylamide gel electrophoresis; DMEM: Dulbecco’s modified eagle medium; DTT: Dithiothreitol; HRP: Horseradish peroxidase; ECL: Enhanced chemiluminescence; cDNA: Complementary deoxyribonucleic acid; GAPDH: Glyceraldehyde phosphate dehydrogenase gene.

## Competing interests

The authors declare that they have no competing interests.

## Authors’ contributions

Conception and design: JRW, YZ, GQJ, DX. Development of methodology: JRW, YZ, GQJ, DX. Acquisition of data: JRW, YZ, GQJ. Analysis and interpretation of data: JRW, YZ, GQJ, DX. Writing and review of the manuscript: JRW, YZ, GQJ, DX. Study supervions: GQJ, DX. All authors read and approved the final manuscript.
